# Selective Oxidation of Cyclohexene over the Mesoporous H-Beta Zeolite on Copper/Nickel Bimetal Catalyst in Continuous Reactor

**DOI:** 10.1021/acsomega.3c10503

**Published:** 2024-06-06

**Authors:** Kanthimathi Tumuluri, Jehad K. Abu-Dahrieh, Kulothungan Mathiyalagan, Aravindan Munusamy Kalidhas, Tamizhdurai Perumal, Santhosh Srinivasan, Varadarajulu Lakshmipathy Mangesh, Nadavala Siva Kumar, Salwa B. Alreshaidan, Kavitha Chandrasekaran, Vijayaraj Arunachalam, Ahmed S. Al-Fatesh

**Affiliations:** † Department of Mechanical Engineering, Koneru Lakshmaiah Education Foundation, Vaddeswaram, Guntur, Andhra Pradesh 522502, India; ‡ School of Chemistry and Chemical Engineering, Queen’s University Belfast, Belfast BT9 5AG, U.K.; § Department of Chemistry, Dwaraka Doss Goverdhan Doss Vaishnav College (Autonomous) (Affiliated to the University of Madras, Chennai), 833, Gokul Bagh, E.V.R. Periyar Road, Arumbakkam, Chennai 600 106, Tamil Nadu, India; ∥ Department of Mechanical Engineering, Faculty of Engineering and Technology, Jain Deemed to Be University, Bengaluru 560004, India; ⊥ Department of Chemical Engineering, College of Engineering, King Saud University, P.O. Box 800, Riyadh 11421, Saudi Arabia; # Department of Chemistry, Faculty of Science, King Saud University, P.O. Box 800, Riyadh 11451, Saudi Arabia

## Abstract

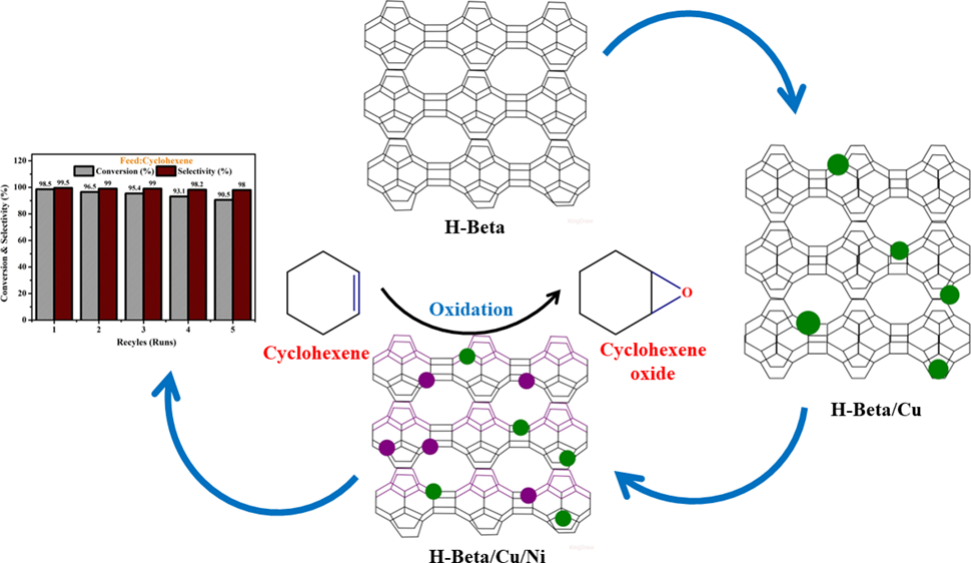

The copper/nickel–metal on commercial H-Beta zeolite supports was synthesized with different wt % (Ni) of 5, 10, 15, and 20, and was used in the cyclohexene epoxidation process. The synthesized catalyst has been used in a continuous reactor for the cyclohexene epoxidation process, with mild conditions and H_2_O_2_ as an oxidant. The catalytic performance was ascertained by adjusting parameters such as the temperature, pressure, WHSV, reaction time, and solvents. The catalytic performance showed the resulting yield in both cyclohexene conversion and selectivity was more than 98.5%. The catalyst’s textural attributes, morphology, chemical composition, and stability were determined using FT-IR, XRD, BET, HR-SEM, and TPD. The most active catalyst among those that were synthesized was evaluated, and the reaction parameters were selected to optimize yield and conversion. The H-Beta/Cu/Ni (15%) catalyst has the best conversion (98.5%) and selectivity (100%) for cyclohexene among the catalysts examined. Cu and Ni(15%) metals were successfully added to the H-Beta zeolite, causing little damage to the crystalline structure and resulting in good reusability over five cycles, as well as little loss of catalytic selectivity. Acetonitrile was the solvent that provided the highest conversion and selectivity among the others. These findings show that H-Beta/Cu/Ni bimetallic catalysts have the potential to be effective epoxidation catalysts. Because of their outstanding conversion and selectivity, the continuous reaction technique used in this work makes them appropriate for industrial production-level applications.

## 1 Introduction

The value of the target compounds for synthetic and commercial purposes has significantly increased since the subsequent products of selective oxidation are attractive synthetic precursors for pharmaceuticals. Epoxides are essential intermediates employed in producing several useful products and fine chemicals.^1^ For the commercial manufacturing of several bulk chemicals, the epoxidation reaction of olefins constitutes an essential and substantial oxidation process.^2^ Cyclohexene oxide, one of the most significant epoxides, is employed in many applications, including creating polymers, dyes, medicines, insecticides, and fragrance goods.^3^ In the industry, the chlorohydrin method and stoichiometric quantities of organic peroxy acids are both often used for selective epoxidation of an alkene C=C double bond. Higher olefins tend to be oxidized with the support of various kinds of heterogeneous metal catalysts incorporating peroxides like H_2_O_2_ or (TBHP) *tert*-butyl hydroperoxide as an oxidant.^4^ Modern organic synthesis has the difficult task of creating and implementing more environmentally friendly and sustainable catalytic epoxidation methods.^5^ Cyclohexene catalytic liquid-phase epoxidation is commercially significant in the formation of cyclohexene oxide, an essential progression in the synthesis of fine chemicals.^6,7^ Numerous studies have investigated the oxidation of cyclohexene in the liquid phase with a predominant focus on the production of cyclohexene-1-one, cyclohexane-1,2-diol, and cyclohex-2-en-1-ol using batch reactors. Only a small quantity of cyclohexene oxide was observed as an intermediate product. The oxidation of cyclohexene using TBHP with CeO_2_@GNFcatalyst resulted in a yield of 89% selectivity with a high conversion of 88%.^8^ The MIL-47(V) catalyst showed differences in product selectivity between liquid-phase and gas-phase operations. Liquid-phase epoxidation resulted in the formation of epoxide, cyclohexenol, and cyclohexenone as primary products.^9^ The Peroxo Phosphotungstic Acid catalyst is great for cyclohexene epoxidation with CH_3_CN and 30 wt % H_2_O_2_. Under mild conditions in 180 min, it achieves 73.2% conversion and 98.2% selectivity. For 89.0% conversion and 94.3% selectivity, it takes longer.^10^ The performance of the Co_3_O_4_/SiO_2_ catalyst in the oxidation of cyclohexene was showed that with an increase in reaction time from 2 to 8 h, the conversion of cyclohexene (37–81%) increased, while the selectivity of 2-cyclohexene-1-one (88–83%) slightly decreased.^11^ SBA-15-DAFO-Pd(II), catalyst was efficient for the selective oxidation of cyclohexene to cyclohexanone, achieving a selectivity of 68.5% for the target product at a conversion rate of 85.5%.^12^

The PVDA_1_-PMo catalyst displayed excellent activity and selectivity, with an optimum cyclohexene conversion rate of 85.7% and selectivity to 2-cyclohexene-1-one of 51.9%.^13^ Mesoporous Ti-AlSi(n) samples exhibited excellent activity in the oxidation of cyclohexene, achieving 100% conversion and selectivity to ketone-alcohol (KA) oil (cyclohex-2-en-1-ol and 2-cyclohexen-1-one) at low temperature and reaction time (35 °C and 30 min).^14^ MNC-10 catalyst displayed selective oxidation of cyclohexene with 90.1% conversion and 92.0% selectivity at the allylic α-position and could be recycled five times without any noticeable decline in activity and selectivity.^15^ The catalytic epoxidation of cyclohexene with H_2_O_2_ over HNb_3_O_8_ samples was conducted in a batch reactor. The yield of allylic oxidation/epoxide-relevant products for HNb_3_O_8_ samples in methanol at 333 K for 1 h was 78% conversion with epoxide-relevant products yield like epoxide (41%), methoxyol (25%), diol (1%), and dial (5%).^16^ MIL-88A(Fe) exhibited high catalytic activity (conversion: 81%), with 70% selectivity for 2-cyclohexene-1-ol in the aerobic oxidation of cyclohexene with O_2_ as the sole oxidant under mild reaction conditions (0.5 MPa of O_2_, 353 K for 8 h).^17^

An improved reaction rate was discovered in the process of the epoxidation of cyclohexene using air as the oxidant. This was achieved without the use of any added catalyst through a continuous flow reactor. The flow process was operated continuously with good operational stability, evaluated by a constant high yield of cyclohexene oxide at 64% with a conversion rate of 73%, resulting in the desired product with high productivity.^18^ The continuous-flow process yielded a significant improvement in reaction time for highly selective epoxide production over the batch process due to the efficient mass transfer between the liquid phase and air. Flow chemistry is emerging as a feasible innovative method for organic synthesis in the academic as well as commercial sectors, offering substantial benefits over conventional batch procedures for organic and inorganic synthesis.^19^ In real terms, multistep flow chemical processes have resulted in an extensive assortment of natural and biologically active compounds.^20^ It is challenging to develop an efficient heterogeneous catalytic system for liquid-phase cyclohexene oxidation due to the two reactive bonds in the cyclohexene molecule. Either the allylic C–H or the olefinic C=C bond can be oxidized.^21^ If epoxidation takes place, then cyclohexene oxide is produced, which is then converted to cyclohexane-1,2-diol via consecutive hydrolysis. Several further oxidation steps, as well as a ring-opening reaction, may proceed.

Zeolite-based catalysts are popular due to their high surface areas, thermal stability, and usefulness as catalyst supports.^22^ Mordenite, erionite, clinoptilolite, chabazite, ZSM-5, Beta, and MCM-22 are the most significant kinds for the industry.^23^ H-Beta zeolite is a phenomenal, high-silica zeolite with a multifaceted overlapping channel pattern, exceptional hydrothermal stability, significant dispersion ability, and minimum steric constraints.^24^ Zeolite H-beta is a versatile catalyst for various industrial applications. It has been used in glycerol dehydration, isomerization, cracking, alkylation, and disproportionation processes.^25,26^

Beta zeolites have been widely researched as a substrate, and Cu^27,28^ or Fe-modified samples have demonstrated outstanding activity.^29,30^ Due to the various functionalities and synergistic effects generated from binary metals, bimetallic cation-exchange zeolite catalysts have recently attracted a great deal of interest. Studies on the dependency of activity, as well as stability in H-Beta zeolite support, are conducted on various bimetallic catalysts consisting of W, Pd, Pt, Rh, Ru,^31−33^ Cu, Ni,^34,35^ Sr, Zr, and Zn.^36−38^ Metal species are well-established to be incorporated into pore-filled substances, such as mesoporous molecules. In comparison to a single metal, the bimetallic alloy exhibits higher activity levels.^39^ These bimetals outperform monometals in terms of their catalytic activity. Supported copper and nickel bimetals are employed in a variety of industrial applications.^40,41^

The objective and aim of this work are to synthesize H-Beta/Cu/Ni bimetallic catalysts that are supported and have varying Ni (wt %) proportions of 5, 10, 15, and 20%. The catalysts will be tested for their catalytic attributes in the epoxidation of cyclohexene, which will be performed in a continuous reactor under liquid-phase conditions, and their nature and reusability.

## 2 Experimental Session

### 2.1 Catalyst Preparation

The catalysts were made using commercially available H-beta zeolite with a 50:50 silica/alumina ratio. The H-beta zeolite was first calcined for 5 h at 550 °C in a furnace, and then it was allowed to cool. Nickel chloride and copper sulfate (Merck) were purchased and used as metal precursors. The sequential wetness impregnation technique was used to produce transition metals loaded on H-beta zeolite. To synthesize bimetallic complexes on an H-beta zeolite. Cu (10%) was initially synthesized by adding 20g of H-beta zeolite that was originally dissolved in 50 mL of water which possessed 7g of CuSO_4_.5H_2_O (a source of copper). The mixture was agitated at 600 rpm for three h at ambient temperature. The mixture was stirred and added to H-beta zeolite; in order to produce the catalysts referred to as H-beta/Cu(10%), substances were initially dehydrated at 110 °C and subsequently calcined over 5 h around 550 °C. With the aid of Nickel chloride as a metal source and the use of the wet impregnation approach, another metallic element, Ni, was integrated into the processed H-beta/Cu(10%) in 4 g. Through the use of this approach, the proportion of nickel ranged from 5 to 20 wt %. The H-Beta/Cu(10%) holding Ni was dried out for 12 h at 110 °C. The sample was subsequently dried before being calcined at 550 °C for 5 h. The H-Beta/Cu (10%)/Ni with Ni (wt %) proportions of 5, 10, 15, and 20% catalysts were synthesized. The ability of a catalyst to catalyze with a range of ratios, which catalyzes the cyclohexene reaction’s epoxidation, was explored.^42,43^

### 2.2 Catalytic Activity Studies

The epoxidation reactions were executed and performed in an incredibly intense pressure, packed bed, down-flow mode stainless-steel processor having an interior diameter of 6 mm. The catalyst, with a size of one to two mm, was picked up and dried for 6 h above 250 °C in CO_2_-free compressed air. The reactor was then subjected to an hour-long cooling process spanning 60 and 110 °C at standard pressure. The feed was then injected after the airflow, the external pressure and temperature settings were set to the optimized limits. The reactions occurred around 60 and 100 °C at WHSV (h^–1^) under a pressure of 10 bar and approximately 3.2 g of preloaded catalyst. The CO_2_-free compressed air and feed with various mole proportions over the cyclohexene epoxidation processes were (20 mmol cyclohexene, 10 mL acetonitrile, and solvent included 25 mmol aqueous water with hydrogen peroxide (H_2_O_2_). The components were analyzed using a gas chromatograph apparatus with a detector for flame ionization using N_2_ as the gas carrier in an SGE BPX70 capillary line.

## 3 Results and Discussion

### 3.1 X-ray Diffraction Analysis (XRD)

**Figure 1:**
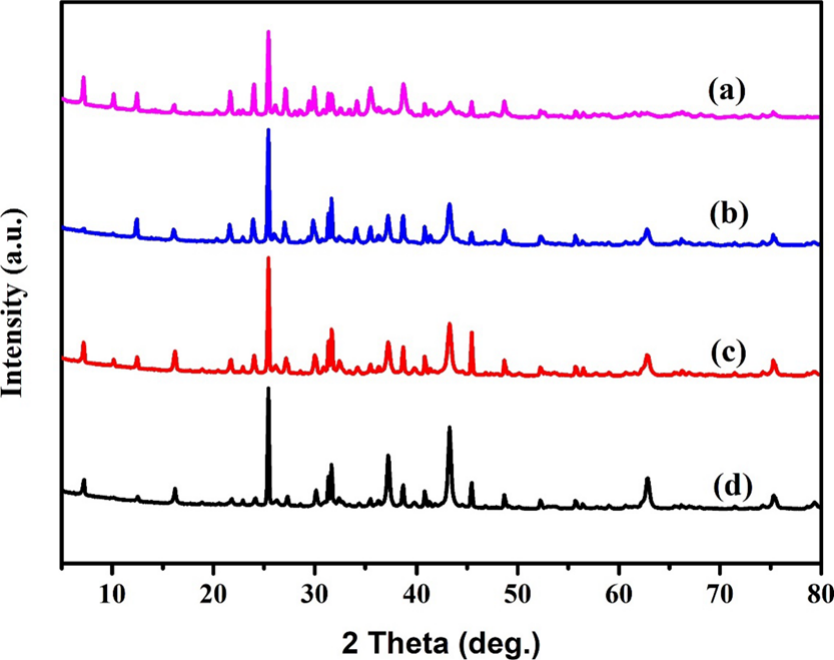
X-ray diffraction patterns of the (a) H-Beta-Cu/Ni(5%), (b) H-Beta-Cu/Ni(10%), (c) H-Beta-Cu/Ni(15%), and (d) H-Beta-Cu/Ni(20%).

The patterns of X-ray diffraction of pristine H-Beta zeolite packed with 10 wt % Copper and various wt % of Nickel (5, 10, 15, and 20%) metals are demonstrated in Figure 1. Two prominent responses at 2θ of 7.8 and 25.3° are appropriate for H-Beta zeolites that possess a well-crystalline morphology in conformity with the literature.^44,45^ The peaks associated with the metal-loaded H-beta zeolite exhibited the same peak as the pristine H-beta zeolite. In another sense, we may reasonably conclude from the graph that metal feeding does not adversely affect the H-beta morphology because of the resemblance in the peaks, which shows that the crystalline phase is retained despite loading H-Beta zeolite with the bimetal.^46^ This could also be attributed to the fact that each metal was an appropriate particle suspended on the H-Beta zeolite. According to the figure, the incorporation of (10%) Cu dosage marginally diminishes the intensity of the distinctive H-beta peaks at 27 and 33°. This is due to the replacement of specific ions (such as H^+^ and Al^3+^) by metal species, which causes some disruption of the zeolites’ structural integrity.^47^ The XRD pattern of the monometallic Cu nanoparticle sample showed three diffraction peaks appearing at 2θ of 43.3, 50.4, and 74.1°, which are characteristic peaks corresponding to the (111), (200), and (220) planes of the face-centered-cubic (fcc) copper (JCPDS 04-0836), respectively. In the monometallic Ni nanoparticle sample, peaks were seen at 44.5, 51.8, and 76.4° corresponding to planes (111), (200), and (220) of the face-centered-cubic (fcc) nickel (JCPDS 04-0850). The bimetallic samples showed diffraction peaks at 2θ of 43.4–43.6, 50.5–50.7, and 74.3–74.6°.^48^ It can be attributed to the (111) plane of the cubic nanostructure of CuNi nanoparticles (JCPDS No. 98–008–7506), which confirms the formation of CuNi NPs. It was observed that after the second metal addition to the monometallic copper-supported catalyst, it exhibits characteristic peaks centered at 2θ = 43.46° the intensity increases with the loading of Ni.^49^ In contrast with the pure H-Beta zeolite, there is a small marginal drop in peak intensity. The peaks of all of the synthesized catalysts exhibit these morphological changes. As the nickel loading was increased, the lines attributed to metallic Ni exhibited higher intensities. Further confirmation that the reduction at 500 °C may not entirely convert Ni^2+^ to metallic NiO or that reoxidation of NiO in the air may contribute to XRD analysis and result in the existence of a small proportion of NiO particles in the catalysts is revealed by a slight shoulder in the XRD pattern of H-Beta-Cu/Ni (15%) and a double line in the XRD pattern of H-Beta-Cu/Ni (20%). Thus, this would imply that both Ni and NiO (Ni-NiO) were incorporated into the catalysts that were utilized in the catalytic reaction. The upsurge in XRD peak intensity associated with rising Ni integrated throughout the catalysts may be attributed to the deterioration in crystallinity or disarrayed behavior induced by the metal incorporation and the consequent rise in pore interference of supporting elements by the overabundance of Ni species. Interestingly, a comparison of the XRD profiles of the endorsed catalysts reveals that the zeolite underwent some amorphization during the catalyst synthesis. It could be anticipated that partial amorphization may occur either during the aqueous solution evaporation process under vacuum at 50 °C or, more probably, during the calcination process.^50,51^
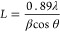
1

The Debye–Scherrer formula was used in order to determine the average particle size of the synthesized catalyst. The average crystallite sizes for H-Beta-Cu/Ni(5%), H-Beta-Cu/Ni(10%), H-Beta-Cu/Ni(15%), and H-Beta-Cu/Ni(20%) zeolites calculated from the (111) diffraction peak were found to be 24.3, 26.6, 32.4, and 34.8 nm, respectively. The average particle size of Beta zeolite increases with increasing Cu/Ni ratio, which is due to the increase in the nickel content on the H-beta zeolite framework.

### 3.2 FT-IR Spectrum

**Figure 2:**
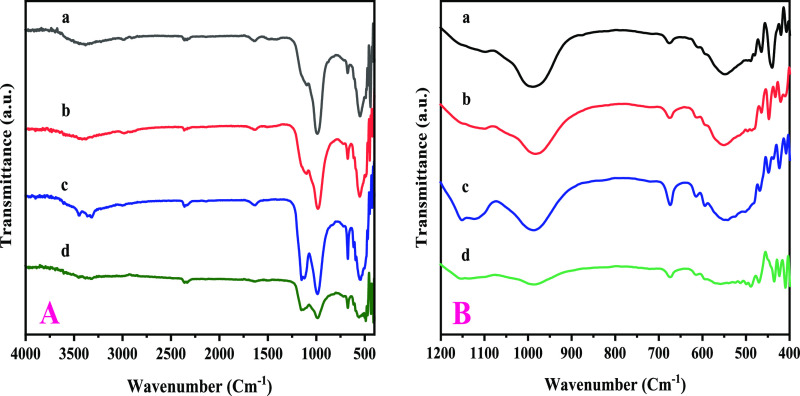
FT-IR spectrum of (a) H-Beta-Cu/Ni(5%), (b) H-Beta-Cu/Ni(10%), (c) H-Beta-Cu/Ni(15%), and (d) H-Beta-Cu/Ni(20%).

The FT-Infrared spectroscopic analysis of H-Beta supported its crystallinity. Zeolitès phase identification in substrates has indeed been accomplished using FT-IR spectroscopy in zeolite chemistry. Interior and exterior bending and stretching vibrations, depicted in Figure 2, that are present predominantly in the FT-IR spectrum exhibited the existence of H-Beta zeolites. At 3453, 1635, 1220, 1150, 796, 553, and 458 cm^–1^, distinctive bands were found.^52^ The T-O bending, external symmetric as well as asymmetric stretching vibrations, and internal asymmetric stretching correspondingly induce the spectrum of absorption at 525, 575,458, 796, 1100, and 1220 cm^–1^ which pertain to siliceous substances. The existence of C_5_ and C_6_-membered rings in the configuration gives the spectrum of absorption at 525 and 575 cm^–1^ their distinctive characteristics as H- beta zeolites.^53^ A recognizable spectrum of absorption at 3640 cm^–1^ is obtained by the separated (Si–O–H) silanol groups, while the spectrum of absorption at 3453 cm^–1^ is associated with the Al–OH Bronsted acid site framework. The MFI-type double five-ring zeolites are responsible for the emergence of a vibration spectrum framework at 553 cm^–1^. Identification of the crystallinity of the resultant product can be done using the 553 cm^–1^ vibrational mode. The Cu/Ni loaded zeolite samples H-Beta/Cu/Ni (5%), H-Beta/Cu/Ni (10%), H-Beta/Cu/Ni (15%), and H-Beta/Cu/Ni (20%) demonstrated that the major band was caused by an asymmetric Si–O–Si stretching mode at 1227 and 1080 cm^–1^ in a typical siliceous material.^54^ Additionally, there was a prominent band: one at 585.8 cm^–1^, which was associated with rocking Si–O–Si, and the other at 800 cm^–1^, which was assigned with symmetric stretching modes in Si–O–Si. (as shown in Figure 1). Additionally, the exclusion of moisture from the ZSM-5 framework during the pellet-making operation with KBr has been associated with the existence of a band at 1635 cm^–1^.

It is imperative to remember that the existence of aluminum results in a decline in the component’s intensity at 950 cm^–1^ which was associated with the Si-(OH) stretching mode. A report claims that the band at about 1092 cm^–1^ is the O–T–O asymmetric stretching vibration (OTO), which is sensitive to the amount of aluminum in the framework. More precisely, this wavenumber proliferated as the proportion of aluminum in the zeolite composition decreased, hence, the variation in this wavenumber reflects a modification that occurred in the molar Si/Al proportions of the framework.^55^

### 3.3 N_2_-Adsorption–Desorption Isotherm (BET)

**Figure 3:**
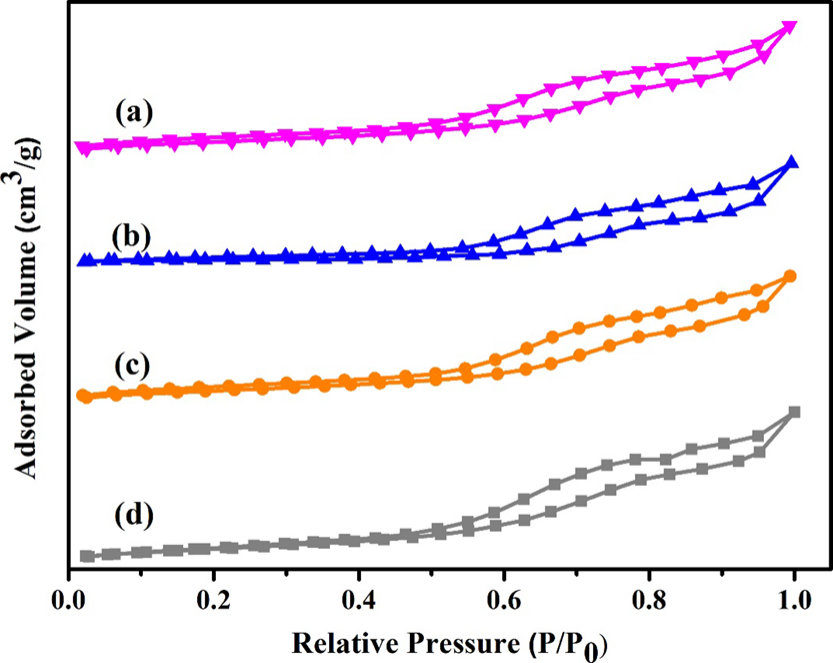
N_2_ adsorption/desorption isotherms of the (a) H-Beta-Cu/Ni(5%), (b) H-Beta-Cu/Ni(10%), (c) H-Beta-Cu/Ni(15%), and (d) H-Beta-Cu/Ni(20%).

The isotherms of N_2_-adsorption–desorption of H-Beta/Cu/Ni(5–20%) catalysts are presented in Figure 3. A 12-ring pore system in 3D made up of H-Beta zeolites possesses channels with two distinct channel types having widths of 6.5 Å × 5.6 and 7.5 Å × 5.7 Å. The isotherms of H-Beta/Cu/Ni(5–20%) exhibit the classic type IV adsorption–desorption isotherm with a hysteresis loop at elevated pressure, suggesting the existence of mesoporous or interparticular void. All the endorsed Cu–Ni catalysts retained the BET-specific surface coverage and volume of pores of the pristine support when Cu and Ni were loaded, with a minimal drop brought on by the slight loading quantities of Cu (10) and Ni (5, 10, 15 and 20). Despite loading copper and nickel, the hysteresis loop exhibits significant distortion, which follows the behavior anticipated on behalf of a substance with unified pores. On H-Beta-Cu/Ni catalysts, measurements of N_2_ absorption at a (*P*/*P*_0_) relative pressure of 0.6–1.0 were also conducted.^56,57^ This is most likely brought on by changes in the layering of the Cu deposits that have built up within the pores. The BET’s total area of coverage and void capacity of Cu/Ni as well as supported H-Beta catalysts are detailed in Table 1. The surface coverage (SA) of the H-Beta/Cu/Ni (5%) catalyst is 442 m^2^/g, while that of the H-Beta/Cu/Ni (20%) catalyst is 401 m^2^/g. Corresponding to this, H-Beta/Cu/Ni (5%) has a wider pore volume (PV) than H-Beta/Cu/Ni (15%), quantifying 0.306 cm^3^/g for H-Beta/Cu/Ni (5) and 0.326 cm^3^/g for H-Beta/Cu/Ni (15%). Component accumulation causes the SA and PV of H-Beta/Cu/Ni(15%) to narrow; the abrupt drop is discernible even at a loading of 10% Cu. The pore-blocking impacts caused by heavy loadings and the oxide accumulation within the pores exacerbate the loss of the exterior area and volume of the pores. The mean pore width inferred from the exterior area and void capacity measurements shows that the pores dramatically narrow at higher H-Beta/Cu/Ni dosage.

**Table 1:** Textural Characteristics of Cu/Ni and Supported H-Beta Catalysts

catalyst entry	surface area (m^2^/g)^a^	*S*_micro_ (m^2^/g)^b^	*S*_ext_ (m^2^/g)	*V*_total_ (cm^3^/g)	*V*_micro_ (cm^3^/g)	*V*_meso_ (cm^3^/g)	acidity (mmol/g)^2^total^c^	LT^d^ peak^c^	HT^e^ Peak^c^
H-Beta-Cu/Ni(5%)	442	380	62	0.306	0.213	0.093	1.01	0.58	0.43
H-Beta-Cu/Ni(10%)	434	366	68	0.320	0.224	0.096	1.12	0.78	0.44
H-Beta-Cu/Ni(15%)	424	348	76	0.326	0.218	0.108	1.36	0.90	0.46
H-Beta-Cu/Ni(20%)	401	327	74	0.299	0.185	0.114	1.43	0.95	0.48

a Measured by the t-plot method.

b *V*_meso_ = *V*_Total_ – *V*_micro_.

c Total acidity was determined by the standard temperature-programmed desorption of ammonia (TPDA) method.

d LT = low temperature.

e HT= high temperature.

### 3.4 HR-SEM Images

**Figure 4:**
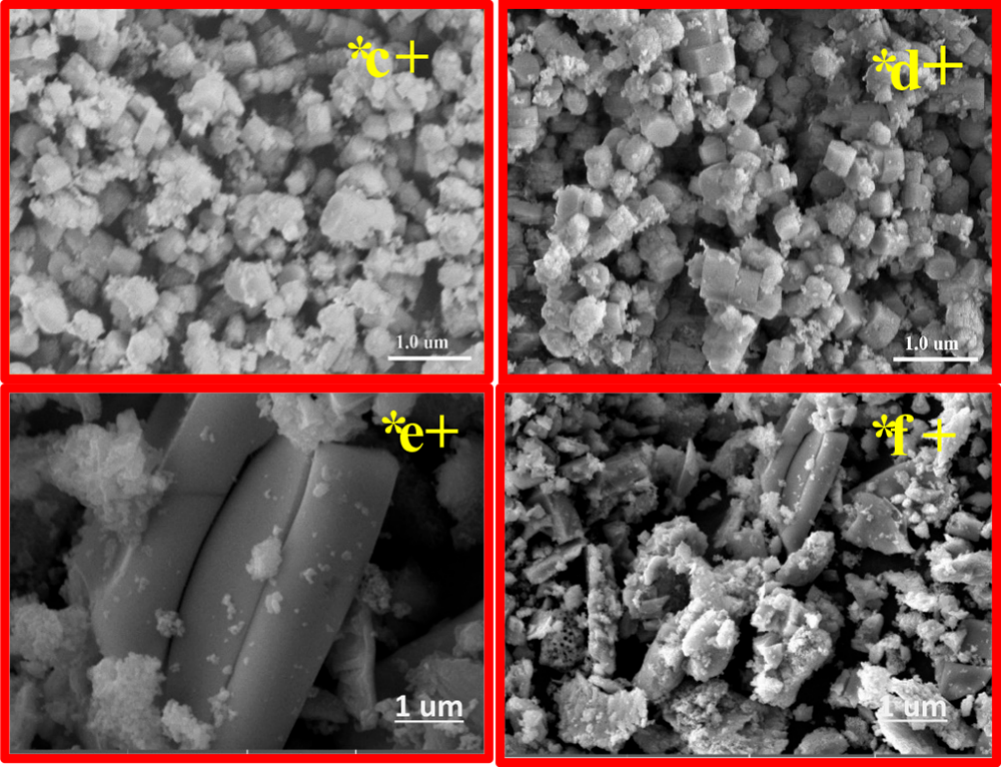
HR-SEM images: (a) H-Beta-Cu/Ni(5%), (b) H-Beta-Cu/Ni(10%), (c) H-Beta-Cu/Ni(15%), and (d) H-Beta-Cu/Ni(20%).

The high-resolution scanning electron micrographs depicted the morphology of the H-beta loaded with copper and various wt.% of Nickel catalysts are shown in Figure 4. On closer inspection, the catalysts’ uniformity and porosity are apparent. Agglomeration results in distinct crystal forms and sizes for the H-Beta/Cu/Ni (5%) catalyst and H-Beta/Cu/Ni (10%). Uniform spherical crystals with a cubic shape were exhibited by the H-Beta/Cu/Ni (10%) catalyst.^58^ The H-Beta/Cu/Ni (15%) and H-Beta/Cu/Ni(20%) catalysts exhibited rod-like crystals. Some crystals seemed to have an octahedral geometry and integrated to produce agglomeration particles. Most crystals with an apparent polygonal morphology are composed of an aggregation of particles with stacking booklet type and pseudohexagonal crystals. Although each edge was extended into a rectangular plane, the overall shape was still a conventional cube. According to this outcome, the surface of this H-Beta zeolite featured a uniform dispersion of elemental copper and nickel. Indicating the catalyst’s porous nature and the H-Beta zeolite’s successful function as a support material, Ni particles were evenly distributed throughout the catalyst, and hardly any substantial Ni particle sizes could be observed on the surface. Based on previous research, the copper particle cannot be clearly spotted owing to the tiny size of the particles of copper metals.^59^ Metal dispersion is capable of being kept low while the metal loading is minimal or moderate.^60^ Furthermore, as nickel loading and nickel crystal growth in the catalyst, the size of the copper crystallites decreases. The distortion is caused by the presence of nickel, which causes an increase in grain size (%).^55,61^ Therefore, it is hypothesized that Ni permeates the zeolite’s porous environment, where the catalytic reforming transition occurs.

### 3.5 Temperature-Programmed Desorption (NH_3_-TPD)

**Figure 5:**
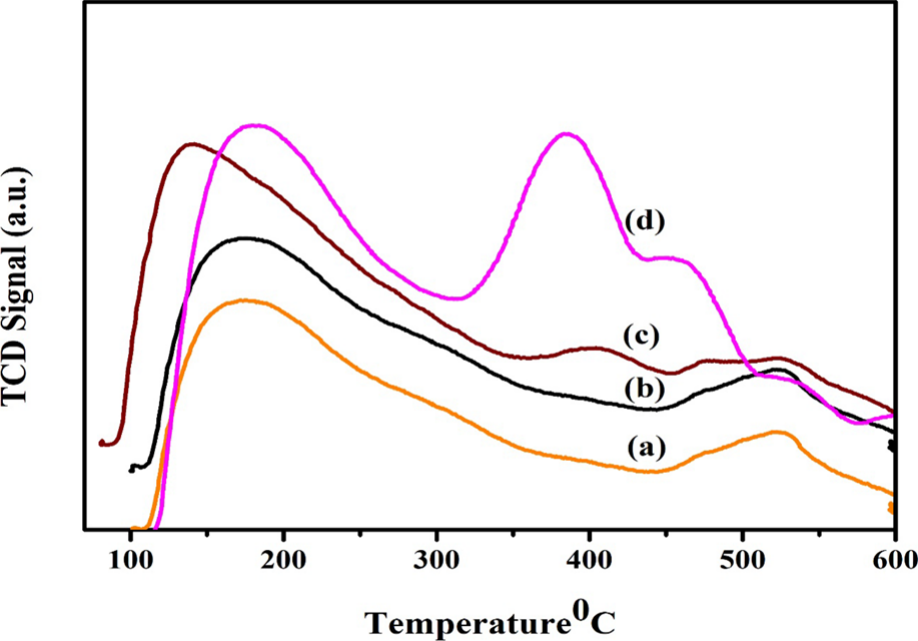
NH_3_- TPD analysis of (a) H-Beta-Cu/Ni(5%), (b) H-Beta-Cu/Ni(10%), (c) H-Beta-Cu/Ni(15%), and (d) H-Beta-Cu/Ni(20%).

The prepared materials’ acidity was evaluated using temperature-programmed ammonia desorption. The stability and quantity of the acid sites present in the catalysts were evaluated using the NH_3_-TPD patterns of the H-beta/Cu (10%)/Ni (5–20%). The catalyst acidity was obtained by using the total quantity of ammonia desorbed, and the level of temperature that resulted in ammonia being eliminated expressed the degree of acid dispersion. Figure 5 and Table 1. display the results. According to the previous research, the value recorded at low temperatures of 200–300 °C is typically associated with NH_3_ deposited on Lewis acid spots, whereas the peak that appeared at 400–500 °C is related to NH_3_ trapped on Bronsted acidic sites.^62^ Based on the intensity of the desorbed ammonia with temperature, weak (less than 200 °C), intermediate (between 200 and 350 °C), and high (beyond 350 °C) areas of acidity were recognized. A prominent desorption signal which seemed to have divided into two halves was apparent in the catalysts, with the weak acid sites portion centered about 150–250 °C and the powerful acid sites (H-Beta zeolite catalyst, 350–450 °C) portion. As the quantity of Ni metal (5–20%) increases, the acidity on the surface rises. H-Beta/Cu/Ni(20%) also showed an additional peak of ∼450 °C, Figure 5 showcases the ammonia desorption from the areas of strong acids coupled with the Al atoms in the framework. The catalyst H-Beta/Cu/Ni(5–20%) contains both weak and strong acid sites in it. The samples’ acidity rises in the order exhibited below: H-Beta-Cu/Ni(5%) < H-Beta-Cu/Ni(10%) < H-Beta-Cu/Ni(15%) < H-Beta-Cu/Ni(20%).

## 4 Catalytic Activity and Reaction Parameter Optimization

### 4.1 Selective Oxidation of Cyclohexene

In continuous reaction circumstances, a variety of H-Beta zeolites were used to selectively oxidize propylene glycol (PG) on copper/nickel samples (Ni molar ratio = 5, 10, 15, and 20%). The influence of WHSV, solvent, and time parameters was improved to maximize propylene glycol (PG) conversion and hydroxyacetone (HA) selectivity by examining the impact of variations in reaction parameters such as the reaction temperature (60–100 °C) and reaction pressure. Furthermore, the most active sample of H-Beta/Cu(10%)/Ni(15) was chosen for reusability testing in the continuous reactor, utilizing optimal parameters. Figure S3 shows the gas chromatographic analysis of the oxidation of propylene glycol.

In the following continuous reaction circumstances, a range of H-Beta/Cu/Ni(5–20%) samples were used for selectively oxidase cyclohexene. To improve the conversion of cyclohexene and the selectivity of cyclohexene oxide, researchers have investigated the influence of adjusting variables related to the reaction which include the temperature of the reaction (60–100 °C), reaction pressurization, the impact of WHSV, solvent, as well as reaction duration aspects. Additionally, the most significantly active H-Beta/Cu/Ni(15%) sample was adopted in the reusable functionality experiment, utilizing the best reactor conditions. As the nickel loading increased, the rate of cyclohexene conversion increased. It was observed that when Ni loading was 20 wt %, the Cyclohexene conversion rate reached 87.5%. However, after a 6 h catalysis reaction by H-Beta-Cu/Ni(15%), the cyclohexene conversion rate was decreased, suggesting that the catalytic activity was reduced. A decrease in catalyst activity may be caused by excessive Ni loading, which increases the Ni particle size and decreases its specific surface area. The large size of the Ni particles would increase coke formation, thereby reducing catalytic activity. Ni particles on the surface of the catalyst were reported to agglomerate as the active metal loading increased, reducing the catalyst’s resistance to carbon deposition and inactivating it.

### 4.2 Outcome of the Catalyst

**Figure 6:**
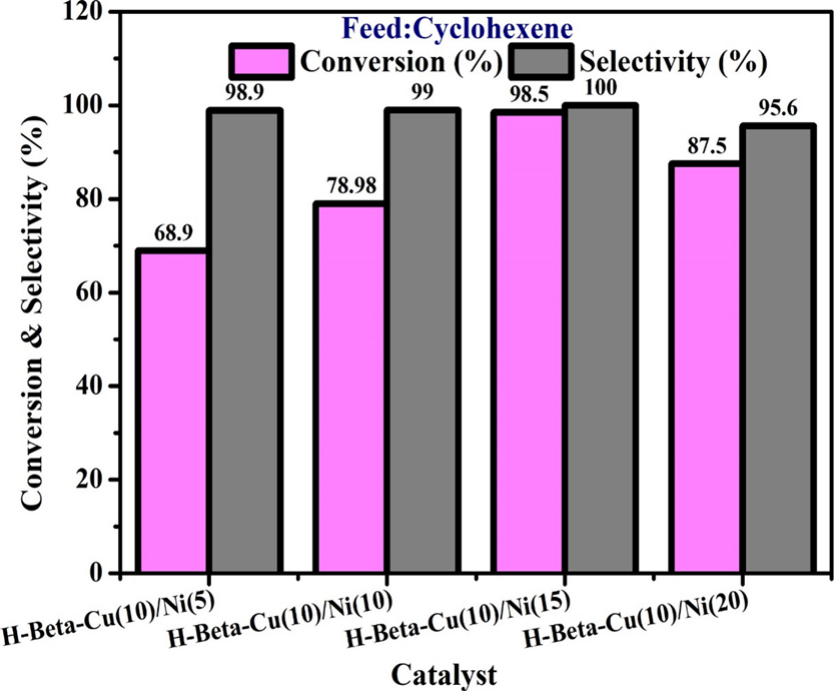
Effect of catalysts on cyclohexene conversion using (a) H-Beta-Cu/Ni(5%), (b) H-Beta-Cu/Ni(10%), (c) H-Beta-Cu/Ni(15%), and (d) H-Beta-Cu/Ni(20%) (reaction conditions: cyclohexene: H_2_O_2_ (1:1), solvent acetonitrile 5 mL, catalyst amount 3.2 g, Pressure 10 bar, Temperature 90 °C).

To achieve the optimum catalytic results, the impact of several parameters on the catalytic capability of H-Beta-Cu/Ni, the most active catalyst, was examined. The four primary products from the oxidation of cyclohexene are 2 cyclohexene-1-oxide, 2 cyclohexene-1-one, 2 cyclohexene-1-hydroperoxide, and 2 cyclohexene-1-ol. The molar proportion of H-Beta zeolite on the copper/nickel bimetal catalyst had a negative impact on the cyclohexene conversion and its product selectivity. Figure 6 demonstrates how the H-Beta zeolite/copper/nickel bimetal catalyst affects the selectivity and conversion of cyclohexene oxidation. Experiments were conducted using a synthesized catalyst for the cyclohexenès conversion and its product selectivity with a 1:1 molar proportion of H_2_O_2_ at 90 °C, and 5 mL of acetonitrile was employed as the solvent. The H-Beta zeolite on the Copper/Nickel bimetal catalyst exhibited the maximum effort, and the preference for 2-cyclohexene-1-one was minimal. This catalytic activity grew steadily as the catalyst’s nickel concentration boosted, and the 2-cyclohexene-1-one selectivity increased. Although H-Beta zeolite on copper/nickel bimetal catalyst revealed the optimal selectivity to 2-cyclohexene-1-one at a molar ratio of 2:3(10%)/(15%), which was nearly 100%. The conversion and selectivity of the H-Beta zeolite on copper/nickel bimetal catalyst considerably decreased when Ni(20%) was raised further. The outcomes propose that proper Cu and Ni wt.% are imperative for the catalyst to function satisfactorily. On the other hand, the existence of a significant number of active sites influences the efficiency of the catalytic system in addition to the acidity of the solids. Based on these discoveries, it can be concluded that the H-Beta zeolite on Copper/Nickel bimetal catalyst could be a useful catalyst in various situations where selective activation of the allylic position is required without changing the double bond. H-Beta zeolite on Copper/Nickel bimetal catalyst’s molar ratio is categorized in the following sequence based on its greater conversion and selectivity: H-Beta/Cu/Ni (20%) < H-Beta/Cu/Ni (5%) < H-Beta/Cu/Ni (10%) < H-Beta/Cu/Ni (15%). To further analyze catalytic activity, H-Beta/Cu/Ni (15%) was optimized.

### 4.3 Effect of Processing Temperature

**Figure 7:**
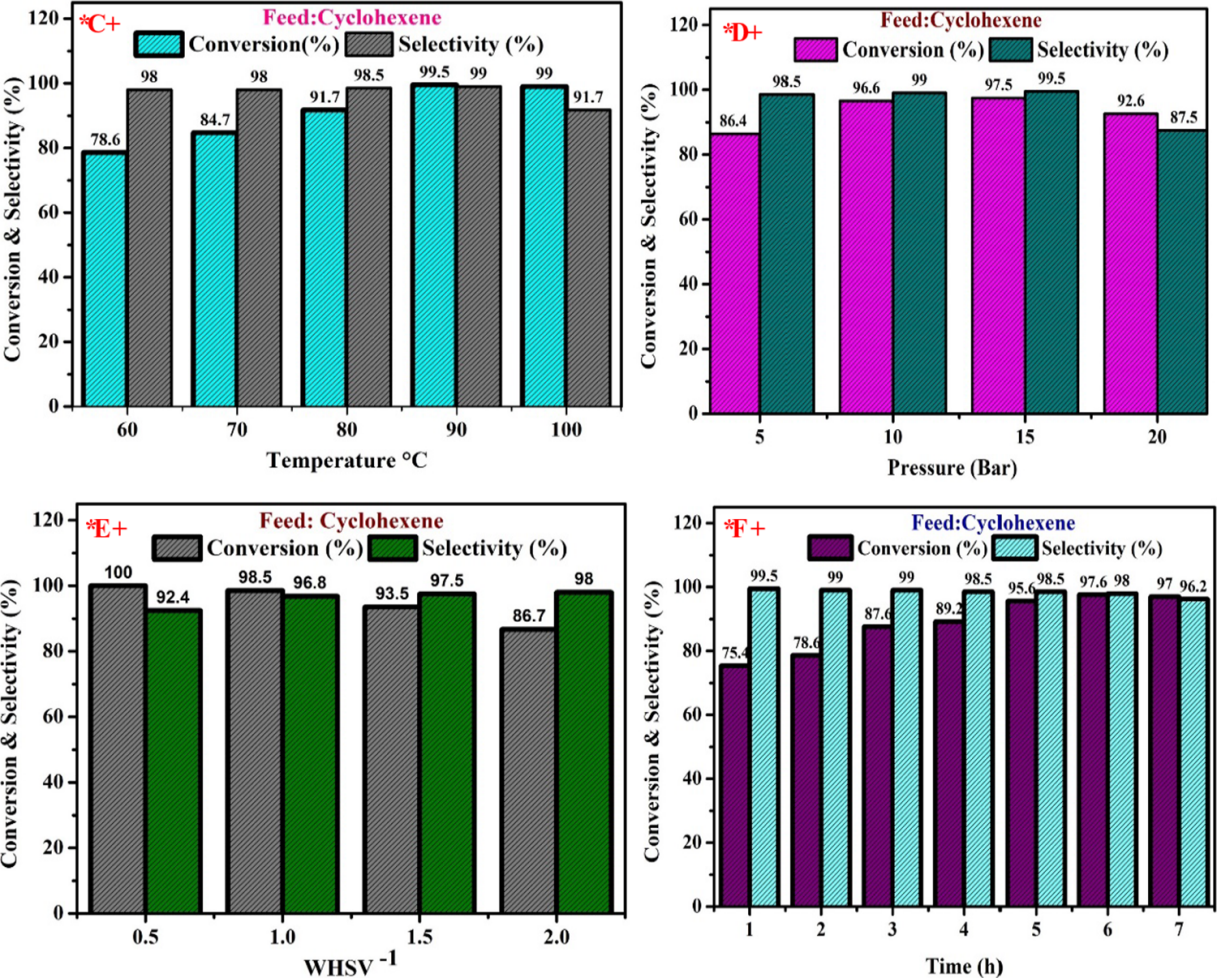
Conversion and Selectivity of Cyclohexene by varying the reaction parameters.

To understand how temperature affects the cyclohexene conversion and its product selectivity, the cyclohexenès specific oxygenation was processed in the range of 60–100 °C. The reaction was carried out using H-Beta zeolite on a Copper/Nickel bimetallic synthesized catalyst for the cyclohexenès conversion and its product selectivity with 1:1 molar proportion of H_2_O_2_ at 90 °C, as well as 5 mL of acetonitrile being employed as the solvent. Figure 7A shows how the reaction temperature affects the oxidation of cyclohexene. When the reaction was run at 60 °C, the conversion of cyclohexene was only 78.6%. When the identical operation is carried out at 70 °C, the conversion of cyclohexene dramatically increases to 84.7%. It is generally agreed upon that reactant conversion will occur more rapidly as the temperature rises. However, it became obvious that as the temperature increased to 80 °C, the conversion of cyclohexene rose somewhat, attaining a maximum of 99.5% at 90 °C. The conversion % gradually declines as the temperature rises further. However, at 60 °C, the selectivity rate reached 98%. At a temperature of 90 °C, it attained a maximum of 99%. However, the percentage of selectivity dramatically drops to 91.7% after a temperature increase of 60 °C from 90 °C. It demonstrates that the distribution of the products was impacted by the reaction temperature. At 60 °C, While the 2-cyclohexene-1-ol̀s selectivity declined, the cyclohexene-1-onès selectivity attained 98%, with temperature, and remained almost unchanged at 70 °C.^63^ This is caused by the remaining overoxidation of byproducts, primarily CO and CO_2_. Therefore, 90 °C concluded as the optimized temperature for the reaction in terms of transformation and selectivity.

### 4.4 Consequences of Pressure

The cyclohexenès conversion and its product selectivity influenced by the subjective pressure is exhibited in Figure 7B. The reaction was carried out using H-Beta zeolite on a Copper/Nickel bimetallic synthesized catalyst for the cyclohexenès conversion and its product selectivity with 1:1 molar proportion of H_2_O_2_ at 90 °C, as well as 5 mL of acetonitrile being employed as the solvent. Cyclohexene conversion rises from 86.4% under 5 bar to 97.5% at 15 bar at 90 °C in the presence of an H-Beta/Cu/Ni (15%) catalyst (Figure 7B). Additionally, the cyclohexenès conversion dropped from 97.5% (under 15 bar) to 92.6% (under 20 bar) due to the H-Beta/Cu/Ni (15%) catalyst’s presence. As shown in Figure 7B, the conversion of cyclohexene ascended initially as the applied pressure varied from 5 to 15 bar; the cyclohexene selectivity percentages shown in Figure 7B replicate the achieved conversion percentage result. H-Beta/Cu/Ni (15%) catalyst promotes the cyclohexenès selectivity from 98.5% at 5 bar to 99.5% at 15 bar. The selectivity percentage likewise rapidly decreases to 87.5% at 20 bar, just like when the conversion percentage occurred. According to the aforementioned findings, raising the operational pressure to 10 bar exhibited a significant influence on cyclohexene’s selectivity and conversion. The intermediate product’s percentage of selectivity initially went up and then dropped as the reaction proceeded at operational pressure between 5 and 15 bar. It occurred because the dissociation of the intermediate products is accelerated by increased pressure. However, because of the increased product oxidation, the extensive pressure had no bearing on the choice concerning cyclohexene transformation and selectivity. The results indicate that this may be the case because higher CO_2_ pressure led to comparatively low cyclohexene concentrations. As a result, the level of transformation of cyclohexene decreased. Therefore, 10 bar concluded as the optimized reaction pressure for the reaction in terms of cyclohexenès conversion and its product selectivity.^64^

(A) Effect of temperature conversion and selectivity of cyclohexene. Reaction conditions: cyclohexene: H_2_O_2_ (1:1), solvent acetonitrile 5 mL, catalyst amount 3.2 g (H-Beta-Cu/Ni(15%)), pressure 10 bar. (B) The effect of pressure conversion and selectivity of cyclohexene. Reaction conditions: cyclohexene: H_2_O_2_ (1:1), temperature (90 °C), solvent acetonitrile 5 mL, catalyst amount 3.2 g (H-Beta-Cu/Ni(15%). (C). The Effect of the WHSV conversion and selectivity of cyclohexene. Reaction conditions: cyclohexene: H_2_O_2_ (1:1), temperature (90 °C), solvent acetonitrile 5 mL, catalyst amount 3.2 g (H-Beta-Cu/Ni(15%), pressure 10 bar. (D). Effect of time on stream on the catalytic activity of cyclohexene. Reaction conditions: cyclohexene: H_2_O_2_ (1:1), temperature (90 °C), solvent acetonitrile 5 mL, catalyst amount 3.2 g (H-Beta-Cu/Ni(15%), pressure 10 bar.

### 4.5 Effect of WHSV

The weight hourly space velocity (WHSV) is obtained by dividing the reactant mass by the catalyst̀s mass. Figure 7C illustrates how the selectivity and conversion of cyclohexene epoxidation are impacted by the inverse weight hourly space velocity (1/WHSV). The reaction was carried out using H-Beta zeolite on a Copper/Nickel bimetallic synthesized catalyst for the cyclohexenès conversion and its product selectivity with 1:1 molar proportion of H_2_O_2_ at 90 °C, as well as 5 mL of acetonitrile being employed as the solvent. Cyclohexene conversion (%) reached 100% in the presence of H-Beta/Cu/Ni (15%) catalysts at WHSV h^–1^ concentrations of 0.5. The conversion rate drops off a little (2% drop-off) at 1 WHSV h^–1^. The conversion rate dropped by approximately % for each every 0.5 WHSV h^–1^ increase (to 1.5 WHSV h^–1^). The ratio of H-Beta/Cu/Ni (15%) declined to a higher degree than that of the other catalysts at 2.0 WHSV h^–1^, dropping from 93.5 to 86.7%. Figure 7C showed that from 0.5 WHSV h^–1^ to 2 WHSV h^–1^, the cyclohexenès conversion rose initially before sharply decreasing. Figure 7C shows this accomplished conversion% result next to the cyclohexene selectivity %. Cyclohexene selectivity (%) rises from 92.4% (at 0.5 WHSV h^–1^) to 98% (at 2.0 WHSV h^–1^) at 90 °C in the existence of H-Beta/Cu/Ni (15%) catalysts. According to the above-mentioned outcomes, the WHSV h^–1^ was raised from 0.5 to 2.0, which decreased the % of cyclohexene’s conversion and increased the % of its product selectivity. The conversion rate plummeted by roughly about 5% for each every 0.5 WHSV h^–1^ increase (to 1.5 WHSV h^–1^). The ratio of H-Beta at 2.0 WHSV h^–1^ arises from the limited concentration of reactant at minimal WHSV h^–1^ and the rising reactant proportion at higher WHSV h^–1^.^65−67^

### 4.6 Effect of Reaction Time

Figure 7D illustrates the analysis of the influence of processing duration on cyclohexenès conversion and its product selectivity using H-Beta/Cu/Ni (15%) at 90 °C. The reaction was carried out using H-Beta zeolite on a Copper/Nickel bimetallic synthesized catalyst for the cyclohexenes conversion and its product selectivity with 1:1 molar proportion of H_2_O_2_ at 90 °C, as well as 5 mL of acetonitrile being employed as the solvent. The substance’s selectivity and conversion % progressively changed over the seven-hour reaction of the experiment. When the amount of contact duration is increased from 1 to 6 h while H-Beta/Cu/Ni (15%) catalysts are in existence, the cyclohexenès conversion increases from 75.4 to 97.6%. The rate of conversion slightly declines as the contact time is extended. As the amount of time grew, the conversion rate decreased. Similar to this, 99.5% selectivity was reached by H-Beta-Cu/Ni(15%) after 1 h. From 1 to 6 h, the selectivity percentage decreases somewhat (1–3% drop-off). With every hour that passed (at 7 h), the overall selectivity percentage decreased by 4% or more. The selectivity% also shows a similar pattern. The results mentioned above demonstrate that selectivity and the rate of cyclohexene conversion tend to decrease as the processing time increases. At the start of the reaction, selectivity increased for the intermediate product before decreasing. The selectivity to 2-cyclohexene-1-one was almost unaltered, It is obvious that during the course of 6 h, the conversion of cyclohexene rose steadily. As the reaction time reached 6 h, the generation of byproducts began to reduce the selectivity to 2-cyclohexene-1-one.^68^

### 4.7 Effect of Different Solvents

**Figure 8:**
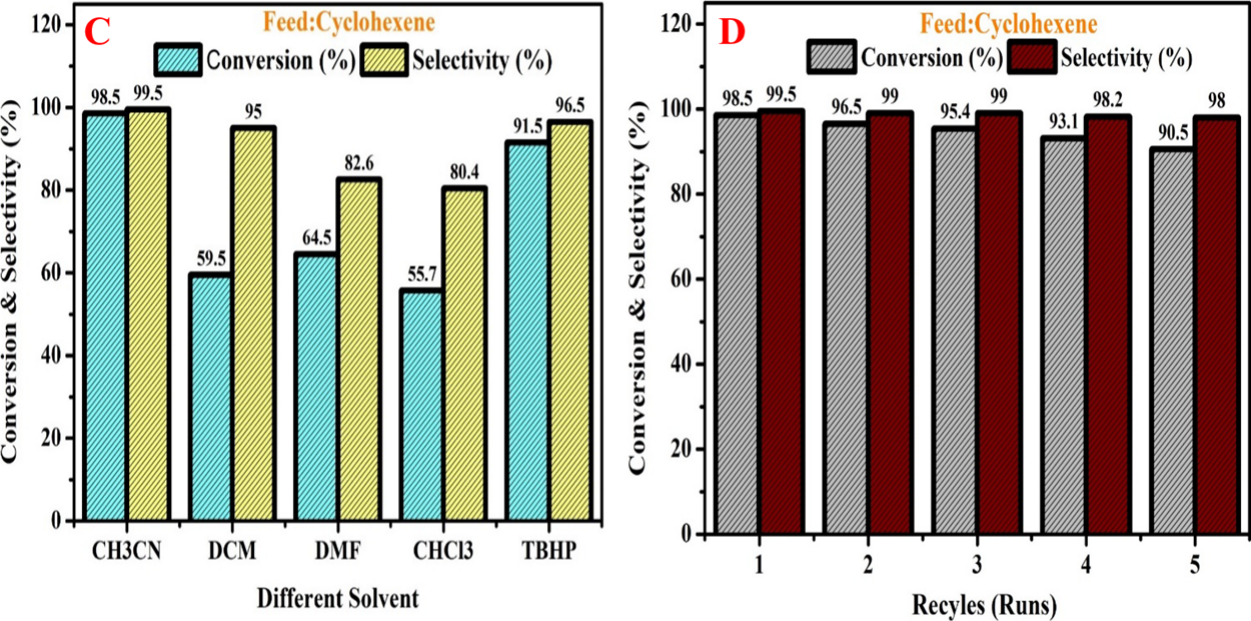
(A) Effect of the solvent on cyclohexene conversion and regeneration studies of cyclohexene oxidation using the H-Beta-Cu/Ni(15%) catalyst. (B) Catalytic recycles of selective oxidation of cyclohexene conversion and selectivity over the H-Beta/Cu/Ni (15%) Catalyst (Reaction conditions: cyclohexene: H_2_O_2_ (1:1), temperature (90 °C), solvent 5 mL, pressure 10 bar, catalyst amount 3.2 g).

The solvents frequently have a large impact on reactions. The results of this assessment of the effects of various solvents on the cyclohexene’s catalytic oxidation over the H-Beta/Cu/Ni (15%) catalyst are shown in Figure 8A. Concerning cyclohexene conversion and selectivity, the catalyst was handled at a molecular ratio of 1:1 H_2_O_2_ to reactant at 90 °C and 10 bar processing pressure. Conversion and its selectivity percent are raised in acetonitrile as a supporting solvent. For cyclohexenès oxidation, it is extremely challenging to regulate the choosiness of the products due to the existence of the two active groups of the C–H bond at the allylic site as well as the C=C bond because when the C–H bond is oxidized, 2-cyclohexene-1-ol, cyclohexene hydroperoxide exposure will be generated since as the C=C bond is oxidized, cyclohexene oxide, cyclohexanol, cyclohexanone, cyclohexanediol, and dialdehyde will be produced. The investigation’s most successful solvent, acetonitrile, improves 2-cyclohexen-1-onès conversion and its product selectivity while DCM, DMF, CHCl_3_, and TBHP are less effective. It occurred by the solvent’s electrostatic attraction, the substrates’ solubility, and reactive oxygen species. In the following sequence, these catalysts tend to be less efficient at oxidizing cyclohexene in various solvents: Chloroform, dichloromethane, TBHP, DMF, and acetonitrile. The polarity of the solvent significantly affects how reactive cyclohexene is. Acetonitrile and TBHP have higher activity as a result of their reduced viscosities. The abstraction and transfer of hydrogen atoms are greatly influenced by the donor–acceptor interaction between intermediate radical species and the solvent, which in turn regulates their reactivity. For each one of the four categories of solvent, conversion rates and selectivity percent raised with progressive polarity, demonstrating that solvents with relatively high polarities are the most effective for catalytic efficiency.^69^

### 4.8 Recycle Run

Since a catalyst’s ability to be reused is crucial from an industrial and economic standpoint. The stability and repeatability of H-Beta/Cu/Ni (15%) in the cyclohexenès selective oxidation reactions constituted thus the subject of our investigation, and the outcomes were exposed in Figure 8B. Exploration was investigated by manipulating the catalyst with cyclohexene conversion and selectivity at a 1:1 molar proportion of H_2_O_2_ to the reactant with 3.2 g of catalyst at 90 °C under 10 bar pressure. The catalyst demonstrated conversion of 98.5% in the first run, which declined to roughly 96.5, 95.4, 93.1, and 90.5% in the second, third, fourth, and fifth runs, respectively. Similar to this, the first run’s selectivity percentage was 99.5%; subsequent cycles’ selectivity percentages were 99, 99, 98.2, and 98%, respectively. These findings indicated that the solvent washing did not completely remove the adsorbed chemicals that were to blame for the activity decline from the catalyst surface and also contributed to the catalyst’s inevitable loss during the collection process. Additionally, there is a 2–3% decline in conversion percentage on each run, although selectivity is almost unaffected. It demonstrated that even after the fifth regeneration step, the catalyst still has a very high level of effectiveness.

### 4.9 Mechanism of the Reaction

**Figure 9:**
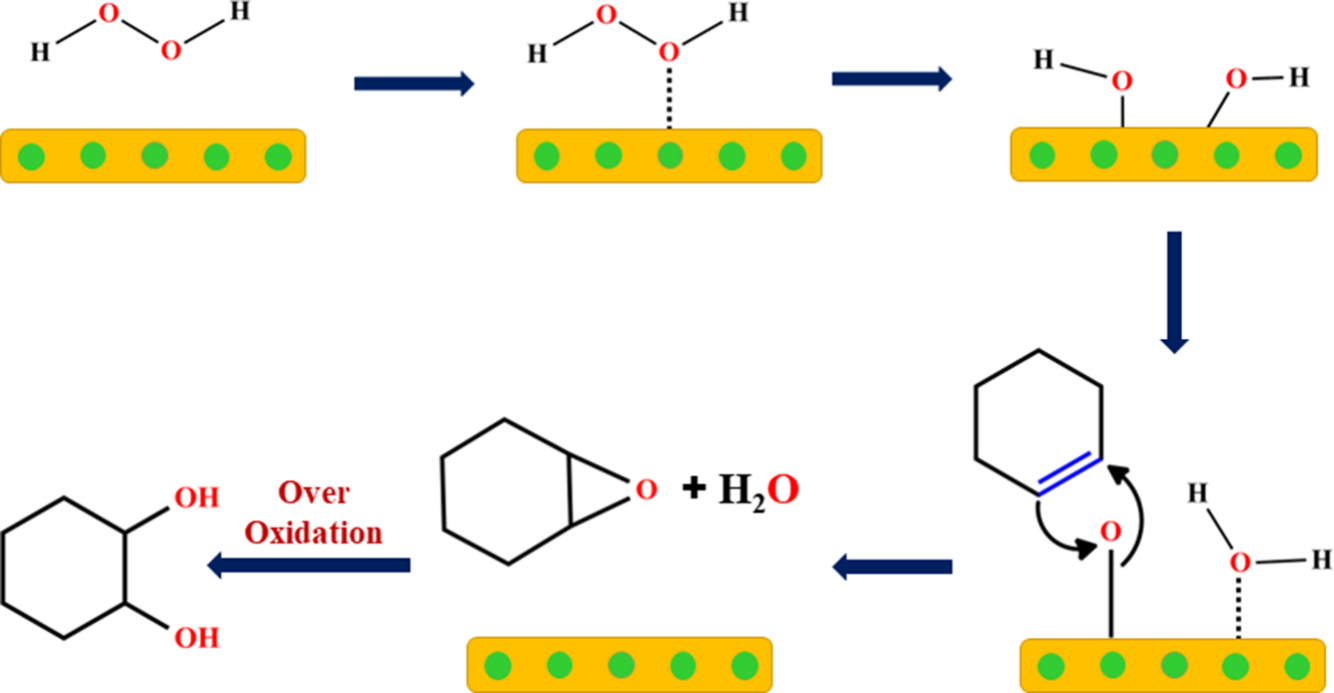
Proposed reaction mechanism for the oxidation of propylene glycol (PG) to hydroxyacetone (HA).

On H-Beta-Cu (10%)/Ni (15%) catalysts, the hypothesized process for selective cyclohexene oxidation is depicted in Figure 9. The selective epoxidation of cyclohexene can be catalyzed by Ni(II) species supported by H-Beta/Cu. The recommended mechanism could be implemented for cyclohexene to cyclohexene oxide consequently taking place in two steps: (1) When Ni (II) species react with the H-Beta surface and H_2_O_2_, dissociation of hydrogen peroxide on the surface of the catalyst (as the reaction is very slow in the absence of catalyst). (2) epoxidation of cyclohexene to form cyclohexene oxide, 3) over oxidation to form cyclohexane 1, 2 diol. The presence of H_2_O_2_ during the formation of cyclohexene oxide epoxidation products indicates a significant impact of the peroxidic oxidant and a different reaction mechanism compared to other oxidants. This can be attributed to the higher O–O bond energy in H_2_O_2_, which increases the activation energy for homolytic cleavage. Additionally, the formation of a complex between H_2_O_2_ and the catalytic metal center is less hindered, allowing for direct oxidation of the olefin by the coordinated H_2_O_2_ and direct transfer of one O atom to the substrate. As a result, epoxidation through coordinated H_2_O_2_ is expected to have higher selectivity and provide access to more valuable epoxidation products. In accordance with these findings, the significance of Cu in Ni-based catalysts has been clarified, providing insights into the complicated interaction between catalyst composition and Selective oxidation of cyclohexene reaction kinetics. In order to maximize cyclohexene selectivity and conversion, the interactions between the catalyst and substrate (cyclohexene) must be carefully balanced. Future research should look into potential ways to minimize the negative effects of Cu in the Ni–Cu catalyst system, such as changing the alloy composition or adding more promoter components to help the reaction process. Furthermore, understanding the relationship that exists between the catalyst’s structural features and its performance in the selective oxidation cyclohexene reaction may help to guide the creation of greater effectiveness and selective catalytic systems.

### 4.10 Catalytic Activity in Comparison

The cyclohexene epoxidation reaction generated in relative percent and reusability were evaluated for comparative catalytic effectiveness under reaction factors, such as conversion and selectivity with the WHSV, pressure, and temperature. Table 2 compares our proposed catalyst with the existing research review for the cyclohexene reaction’s epoxidation. Notably, the catalyst discovered in this study not only continued to be more effective than other catalysts that have been thoroughly addressed in the literature but was also capable of activity. Additionally, the material that has been reported has shown amazing performance in terms of accelerating the cyclohexene epoxidation reaction.

**Table 2:** Catalytic Performances in the Epoxidation of Cyclohexene over Various Catalysts

catalyst entry	catalyst dose (g)	time (h)	temperature (°C)	conversion (%)	selectivity (%)	solvent	ref
Pt/mordenite(40)	2.1	7	160	82.6	87.5	CH_3_CN	(42)
WSn-MFI-a	2.3	9	165	92.3	85.6	CH_3_CN	(43)
hp-polyHIPE-90C		24	140	97.1	97.1	CH_3_CN	(44)
V (IV) Schiff-base/GO	1.05	10	140	95	92.5	CH_3_CN	(45)
10% Ni@CSs	1.05	14	180	98	55	CH_3_CN	(46)
Mo@RGO-2	2.2	9	180	100	93	CH_3_CN	(47)
H-Beta/Cu/Ni(15%)	3.2	6	90	99.5	100	CH_3_CN	this work

## 5 Conclusions

A wet impregnation method was used to effectively create the bifunctional catalyst on H-Beta/Cu/Ni, which is composed of the pores of mesoporous H-Beta catalysts and Cu/Ni bimetals attached to the surface of the pores. The Ni–Cu catalyst was discovered to influence the selectivity of the selective oxidation of cyclohexene; specifically, the impregnation of Cu/Ni dramatically increased the selective oxidation of cyclohexene while decreasing the catalyst’s overall activity. A number of important stages for the selective oxidation of cyclohexene on bimetallic catalysts were proposed, demonstrating that the cyclohexene reaction on Ni–Cu catalysts follows a cyclohexene route, whereby cyclohexene is produced via an associative mechanism. The most effective catalyst among the others is H-Beta/Cu/Ni(15%), which produces a large number of Lewis and Bro̷nsted acid active sites. H-Beta/Cu/Ni(15%) was investigated to study the oxidation of cyclohexene in the liquid phase utilizing acetonitrile as the solvent in a continuous reactor under ideal circumstances for high conversion at 90 °C. When compared to the other catalysts being studied, it had the highest rate of conversion (98.5%) and selectivity (99.5%) rates. Furthermore, even after five consecutive cycles, considerable reusability with just a slight loss of cyclohexene oxide selectivity was achieved in the existence of H-Beta/Cu/Ni (15%). To design and develop more efficient catalysts for epoxidation reactions that can be used in practical applications, it is still necessary to fully comprehend the characteristics of active sites and the unique synergistic effect on the mechanism of bimetallic species. The present catalyst has numerous uses in both industry and educational studies as a result.

## Supporting Information Available

HR-TEM micrographs of H-Beta-Cu/Ni(5–20%), thermal stability of the synthesized catalyst was studied using TGA, and product analyzed using GC analysis of oxidation of propylene glycol (PDF)
