# Supplementary material for: Selective Oxidation of Cyclohexene over the Mesoporous H-Beta Zeolite on Copper/Nickel Bimetal Catalyst in Continuous Reactor
Source: ACS Omega. 2024 Jun 6;9(24):25800–11. doi: 10.1021/acsomega.3c10503 (PMC11191118; doi:10.1021/acsomega.3c10503)
Supplement: Supplementary file 1 — ao3c10503_si_001.pdf [file ao3c10503_si_001.pdf]

Electronic Supplementary data

Selective Oxidation of cyclohexene over the mesoporous H-Beta zeolite on Copper/Nickel bi-metal catalyst in continuous reactor

Kanthimathi Tumuluri[1], Jehad K. Abu-Dahrieh[2*], Kulothungan Mathiyalagan[3], Aravindan Munusamy Kalidhas[4], Tamizhdurai Perumal*[3], Santhosh Srinivasan[3], V.L. Mangesh[1], Nadavala Siva Kumar[5], Salwa B. Alreshaidan[6], Kavitha Chandrasekaran[3], Vijayaraj Arunachalam[3], Ahmed S. Al-Fatesh[5]*

[1]Department of Mechanical Engineering, Koneru Lakshmaiah Education Foundation, Vaddeswaram, Guntur district, Andhra Pradesh522502, India

[2]School of Chemistry and Chemical Engineering, Queen's University Belfast, Belfast, BT9 5AG, UK

[3]Department of Chemistry, Dwaraka Doss Goverdhan Doss Vaishnav College (Autonomous) (Affiliated to the University of Madras, Chennai), 833, Gokul Bagh, E.V.R. Periyar Road, Arumbakkam, Chennai 600 106, Tamil Nadu, India

[4]Department of Mechanical Engineering, Faculty of Engineering and Technology, Jain deemed to be university. Bengaluru

[5]Department of Chemical Engineering, College of Engineering, King Saud University, P.O. Box 800, Riyadh 11421, Saudi Arabia

[6]Department of Chemistry, Faculty of Science, King Saud University, P.O. Box 800, Riyadh 11451, Saudi Arabia

* **Corresponding author.**

Dr.Jehad K. Abu-Dahrieh; E-mail address: j.abudahrieh@qub.ac.uk

Dr. P. Tamizhdurai; E-mail address: tamizhvkt2010@gmail.com

Prof. Ahmed S. Al-Fatesh; E-mail address: aalfatesh@ksu.edu.sa

High-Resolution Transmission Electron Microscopy (HR-TEM)

The size, shape, and dispersion of active metals are measured using high-resolution transmission electron microscopy. Figure S1(a-d) displays HR-TEM micrographs of Ni (5-20 wt.%) and Cu (10 wt.%) impregnated on H-Beta. H-Beta zeolite size and structure vary significantly as a result of alkaline treatment. Figure S1 (a-d) depicts the existence of mesopores. Figure S1 (a) depicts nano-sized H-Beta particles with the inclusion of Ni metal, and the 10% Cu metal is randomly dispersed on the H-Beta zeolite. Figure S1 (b) depicts an uneven shape of mordenite particles containing Ni metal, and the agglomeration is caused by the addition of 10% Cu metal. Figure S1 (c-d) shows that increasing the quantity of metal inclusion results in an obscured surface shape of the H-Beta zeolite. The dark patches indicate that the Ni and Cu metals are evenly scattered across the H-Beta zeolite.

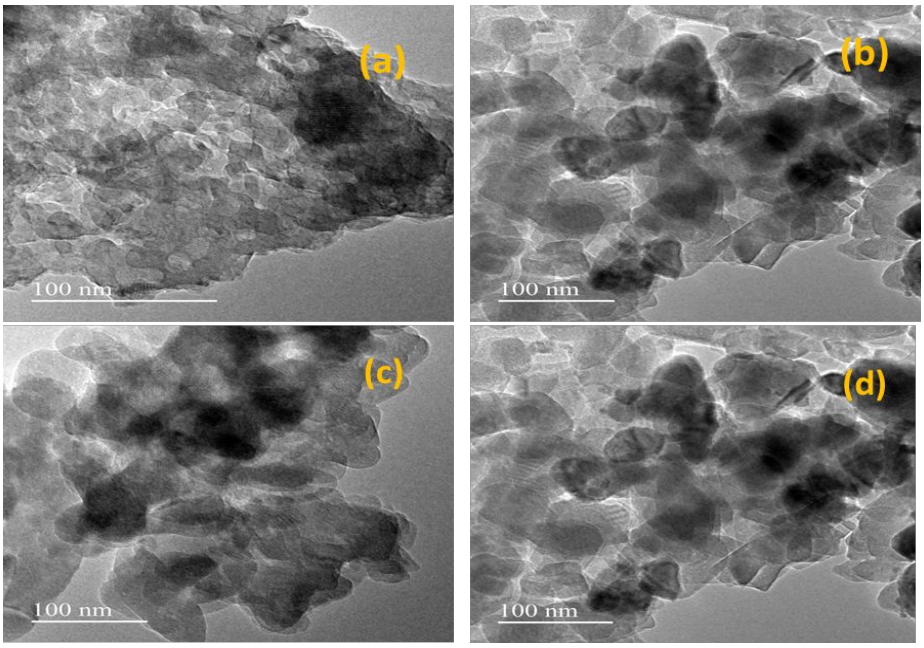

Figure S1.HR-TEM images a) H-Beta-Cu/Ni(5%), b) H-Beta-Cu/Ni(10%) c) H-Beta-Cu/Ni(15%), and d) H-Beta-Cu/Ni(20%).

Thermal gravimetric analysis (TGA)

The thermal stability of the synthesised zeolite was assessed using TGA. Figure.S2 depicts TGA curves for a) H-Beta-Cu/Ni(5%), b) H-Beta-Cu/Ni(10%), c) H-Beta-Cu/Ni(15%), and d) H-Beta-Cu/Ni(20%) samples. The H-Beta-Cu/Ni surfactant has a temperature range of 160-280 °C. The surfactant is removed from the zeolite framework during the process of calcination. The surfactant has been shown to work primarily during the ageing the platform, as it is only stable during this time and will be destroyed by high temperature crystallisation. The elimination of template causes the final weight loss between 320 and 530 °C. All four H-Beta-Cu/Ni samples show a two-phase weight reduction, which corresponds to water loss and organic template degradation. a) H-Beta-Cu/Ni (5%), b) H-Beta-Cu/Ni (10%), c) H-Beta-Cu/Ni (15%), and d) H-Beta-Cu/Ni (20%). H-Beta-Cu/Ni exhibits weight losses of 2.46%, 3.97%, 6.28%, and 10.31%, respectively. At temperatures that are higher (500-700 °C), all samples demonstrate three stages of weight loss. The weight loss for a) H-Beta-Cu/Ni(5%), b) H-Beta-Cu/Ni(10%), c) H-Beta-Cu/Ni(15%), and d) H-Beta-Cu/Ni(20%) zeolites is 6.96%, 7.92%, and 9.67%, respectively. The first weight loss at less than 150 °C is caused by the elimination of water from all four H-Beta-Cu/Ni samples, and the second weight loss is caused by the breakdown, which results in insignificant weight loss due to the complete combustion of every one of the organic templates. Based on the aforementioned data, we conclude that all of the samples are stable at higher temperatures.

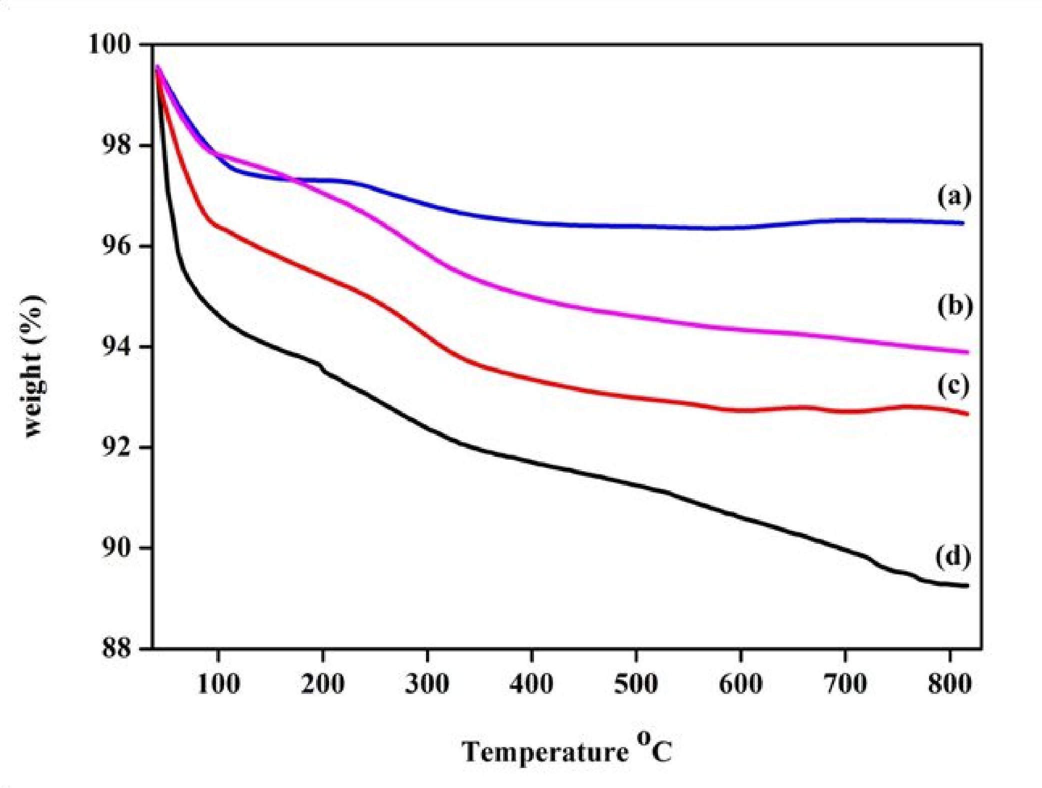

Figure. S2.TGA analysis of as-synthesized zeolite: a) H-Beta-Cu/Ni(5%), b) H-Beta-Cu/Ni(10%), c) H-Beta-Cu/Ni(15%) , and d) H-Beta-Cu/Ni(20%).

Figure S3

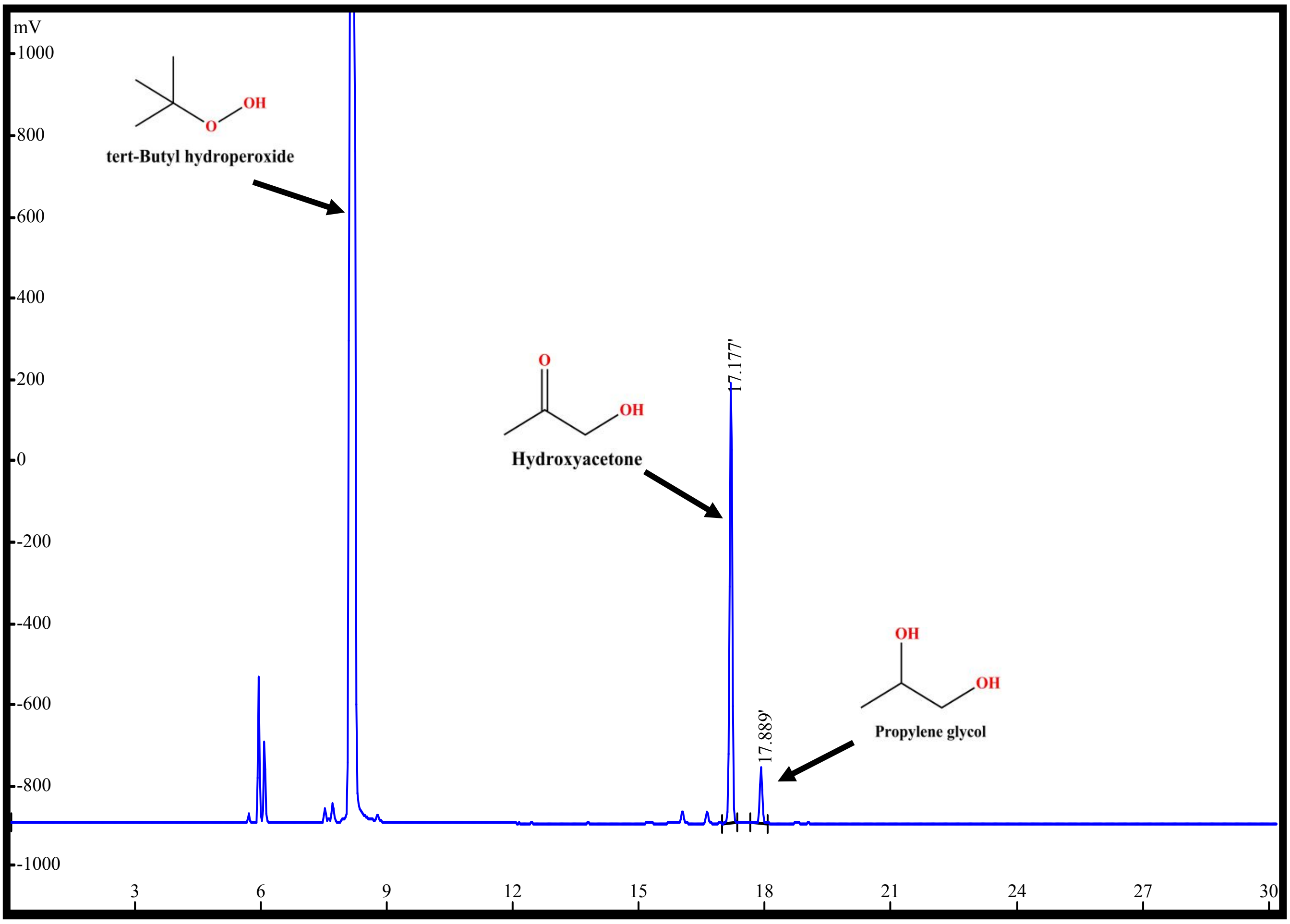

Figure S3. Gas Chromatography analysis of oxidation of propylene glycol
